# Epilepsy in *MT*‐*ATP6* ‐ related mils/NARP: correlation of elettroclinical features with heteroplasmy

**DOI:** 10.1002/acn3.51259

**Published:** 2021-01-21

**Authors:** Laura Licchetta, Lorenzo Ferri, Chiara La Morgia, Corrado Zenesini, Leonardo Caporali, Maria Lucia Valentino, Raffaella Minardi, Daniela Fulitano, Lidia Di Vito, Barbara Mostacci, Lara Alvisi, Patrizia Avoni, Rocco Liguori, Paolo Tinuper, Francesca Bisulli, Valerio Carelli

**Affiliations:** ^1^ IRCCS Istituto delle Scienze Neurologiche di Bologna Full Member of the ERN EpiCARE Bologna Italia; ^2^ Department of Biomedical and Neuromotor Sciences University of Bologna Bologna Italia; ^3^ Neurology Unit Rovigo Italy

**Keywords:** epilepsy, maternally inherited Leigh's syndrome (MILS), MT‐ATP6 MT‐ATP6, neuropathy, ataxia, retinitis pigmentosa (NARP), progressive myoclonic epilepsy (PME)

## Abstract

The study aims to characterize the epilepsy phenotype of maternally inherited Leigh's syndrome (MILS) and neuropathy, ataxia, retinitis pigmentosa (NARP) due to mutations in the mitochondrial *ATP6* gene and to correlate electroclinical features with mutant heteroplasmy load (HL). We investigated 17 individuals with different phenotype, from asymptomatic carriers to MILS: 11 carried the m.8993T> G mutation, 5 the m.8993T> C and one the novel, *de novo* m.8858G> A mutation. Seizures occurred in 37.5% of patients, EEG abnormalities in 73%. We ranked clinical and EEG abnormalities severity and performed quantitative EEG to estimate Abnormality Ratio (AR) and Spectral Relative Power (SRP). Spearman’s rho and Kruskal–Wallis test were used for correlation with heteroplasmy load (HL). HL correlated with disease severity (Rho = 0.63, *P* = 0.012) and was significantly higher in patients with seizures or EEG abnormalities (*P* = 0.014). HL correlated with EEG severity score only for the m.8993T> G (Rho = 0.73, *P* = 0.040), showing a trend toward a positive correlation with AR and delta SPR, irrespective of the mutation.

## Introduction

Mutations in mitochondrial ATP6 gene (*MT‐ATP6*) cause a spectrum of progressive neurodegenerative disorders ranging from MILS (maternally inherited Leigh's syndrome)^1^ to NARP (neuropathy, ataxia, retinitis pigmentosa).^2^ Milder manifestations further broaden the clinical spectrum to include oligosymptomatic cases (incomplete NARP). Different phenotypes can occur in the same family.^3^, ^4^

*MT‐ATP6* encodes the ATP6 subunit of the F1F0‐ATPase (ATP synthase/complex V). The most common mutation is the heteroplasmic m.8993T> G.^5^, ^6^

The wide variability of disease severity and age at onset has been mainly attributed to the degree of heteroplasmy, the higher the percentage of mutant mtDNA, the more severe the phenotype.^1^, ^2^, ^3^, ^4^, ^7^

Epileptic seizures are reported in up to 86% and 44% of patients with MILS and NARP, respectively.^6^ Ictal manifestations range from intractable generalized and focal seizures in MILS^3^, ^4^, ^8^, ^9^ to occasional generalized tonic‐clonic seizures (GTCS) in adult onset‐NARP.^2^ In the latter, epileptiform EEG abnormalities preceding the onset of epilepsy by several years have also been reported,^10^ with potential implications in terms of seizure surveillance.

The disease severity of NARP and MILS appears to be related to the occurrence of seizures, the age at seizures onset and the epilepsy phenotype. However, no studies have explored the association between epilepsy and the heteroplasmy load (HL) of mtDNA mutations so far.

We here characterized the epilepsy phenotype of patients with mitochondrial diseases due to *MT*‐*ATP6* mutations. The electro‐clinical study, extended to asymptomatic carriers, included quantitative EEG (qEEG) spectral analysis in a subgroup. We aimed to provide prevalence esteem of seizures and EEG abnormalities in our case‐series and investigate the correlation of HL with electroclinical and qEEG findings.

## Methods

The study was approved by the Local Ethics Committee (13036).

We investigated patients with MILS or NARP referred to our Institute between 1971 and 2019. All index‐cases and available affected/unaffected relatives underwent clinical and neurophysiological assessment including EEG and/or polygraphic recording. Clinical information on unreachable or deceased family members was collected for pedigree reconstruction. We focused on epileptic phenotype, referring to the ILAE guidelines (www.epilepsydiagnosis.org).

Genetic analysis was performed by direct sequencing of *MT*‐*ATP6*. Since mutant mtDNA shows fairly uniform tissue distribution,^11^ HL was quantified in DNA extracted from blood in all index‐cases and maternal relatives by SNaPshot assay (detailed in Appendix S1).

All individuals carrying *MT*‐*ATP6* mutations were included. Based on clinical and molecular findings we distinguished different affection status, scoring disease severity as follows: 0 = asymptomatic carrier; 1 = incomplete NARP with one symptom of the triad; 2 = incomplete NARP with two symptoms; 3 = NARP; 4 = NARP with encephalopathy and/or transient basal ganglia lesions (labeled “NARP‐MILS”); 5 = MILS as defined by bilateral striatal lesions. EEG patterns were categorized according to a 0‐5 ranking scale, with 0 = normal trace/non‐specific changes and 5 = combination of slow background activity (BA), burst of slow‐waves and spike‐wave discharges (Appendix S1‐S2). Quantitative EEG (qEEG) spectral analysis was performed in all patients who underwent a 19‐electrodes‐digital registration according to a standardized protocol. Abnormality Ratio (AR) and Spectral Relative Power (SRP) of Delta, Theta, Alpha, and Beta band were obtained for each patient (Appendix S1).

Statistical analysis was performed using statistical package Stata SE 14.2. Spearman’s rank correlation (Rho) was used to evaluate the strength and direction of the relationship between HL and continuous variables, whereas Kruskal–Wallis test to compare the median level (and Interquartile Range, IQR) of HL for dichotomic categories. A sub‐group analysis was performed for the m.8993T> G mutation.

## Results

**Figure 1:**
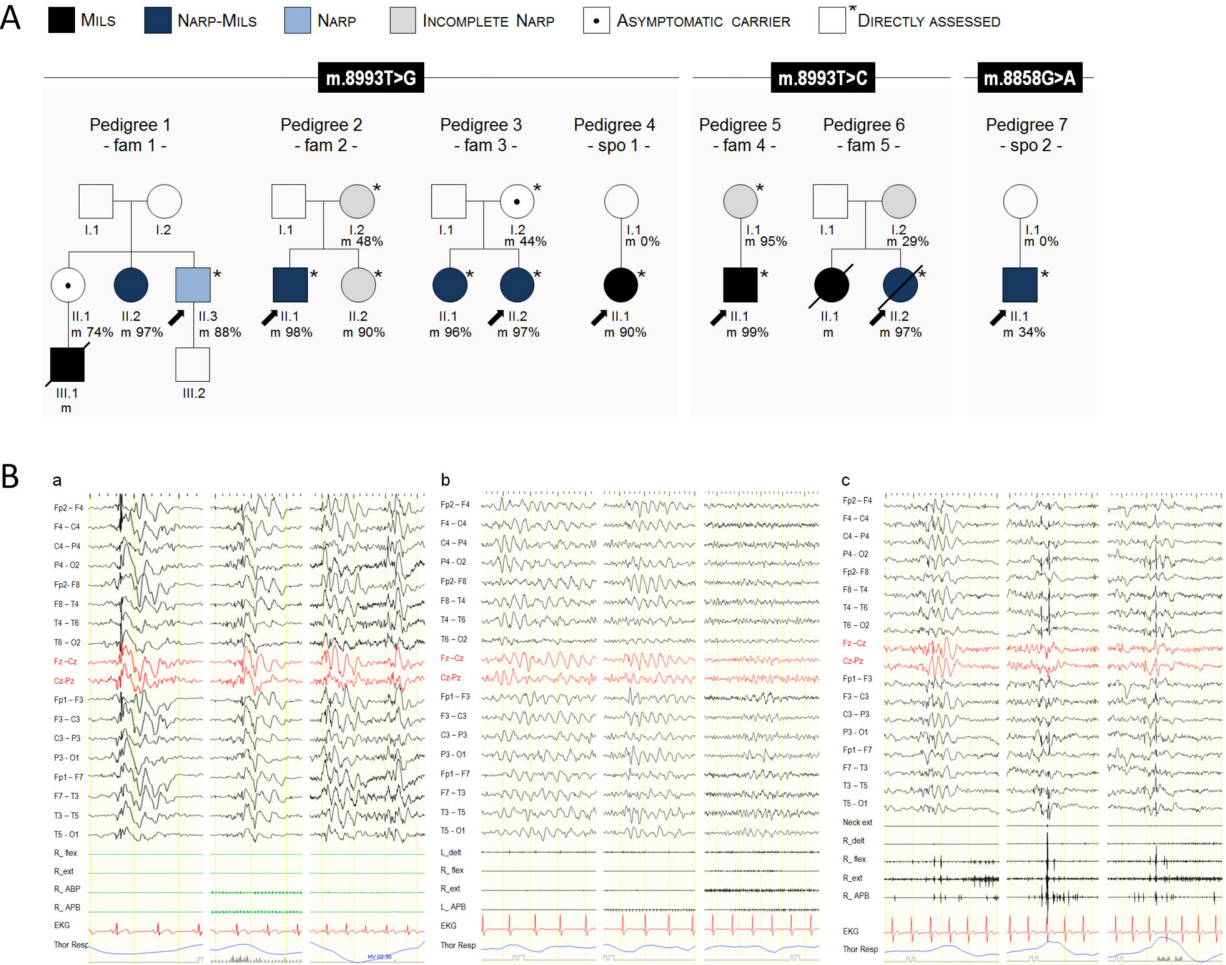
(A) pedigrees of the seven unrelated cases included in the study. (B) EEG and polygraphic recordings. Montage: EEG channels (double banana); polygraphic channels abbreviations: L:left; R:right; delt: deltoid; flex: flexor carpi radialis; ext: extensor carpi radialis; APB: abductor pollicis brevis; neck ext: neck extensors; EKG: electrocardiogram; Thor Resp: thoracic respirogram. **a**: Individual 2/II.2 – incomplete NARP ‐ 8993T> G (HL 90%). Irregular S‐W discharges enhanced by IPS and HV and without clinical correlate. IPS response is only attenuated by blue lenses. **b**: Individual 4/II.1 ‐ NARP‐MILS ‐ 8993T> G (HL 90%). Runs of high‐amplitude, monomorphic slow waves, sometimes sharply‐contoured, diffuse but prevalent over the anterior or central‐parietal regions. Sharp slow waves sometimes with left temporal‐parietal prevalence. **c:** Individual 2/II.1 ‐ PME in NARP‐MILS ‐ 8993T> G (HL 98%). Irregular S‐W discharges, diffuse, sometimes prevalent over the parieto‐temporal fields. Surface EMG shows bursts of erratic myoclonias over the upper limbs explored, often with clear‐cut cortical correlate.

We studied seven unrelated index‐cases with MILS or NARP syndrome (five familial and two isolated) and 10 family members carrying various HL of *MT‐ATP6* mutations. Of the 17 individuals (M/F:5/12), 15 were affected (four with MILS, six with NARP‐MILS, one with NARP, four with incomplete NARP) and two were asymptomatic carriers. Figure 1A shows the pedigrees, three of which previously reported (pedigree 2^4^, ^7^, 3^7^, 6^12^).

Twelve individuals were directly assessed (mean age at last assessment: 41.3 ± 13.6 years; mean follow‐up: 12.2 ± 13.2 years). The remaining five include two children with MILS (pedigree 1/III.1; 6/II.1) deceased at age 3 years following an acute metabolic crisis. All electro‐clinical and genetic findings are detailed in Table 1.

**Table 1:** Electroclinical features of the 17 individuals with *MT‐ATP6* pathogenic variants included in the study

*MTATP6* variant	Phenotype (#)	HL (%) range	Age at onset	Symptom at onset	Other signs/symptoms	Seizure type	EEG/polygraphy	Neuroimaging	ASM
8993T>G	MILS (2)	90‐na	2 m‐ na	1 acute E 2 psychomotor delay	1 cer/pyr, M, ID 1 na (deceased at 3 y)	1 none 1 na	1 sW> post, S‐W 1 na	1 BG, CA 1 na	1 None 1 na (deceased)
	NARP‐MILS (4)	96‐98	birth‐1 y	4 psychomotor delay + seizures (1)	2 severe E, atonia, dyskynesia 2 hypotonia, cer/pyr	1 GTCS, As 1 Ms (PME) 1 GTCS 1 GTCS, M	2 slow BA, sW, S‐W 1 slow BA 1 na	1 BG (t), CA, WM 2 CA 1 na	1 PB, BDZ 1 LEV, CBD+TCH 2 PB
	NARP (1)	88	29 y	1 RP, A	hyp, AF, fat, mig, ext	None	slow BA, sW	1 WM, CA	None
	Incomplete NARP (2)	48‐90	17‐32 y	2 RP	1 N, mig, M, fat; 1 A, mig, M	None	1 S‐W; 1 n/s	1 normal; 1 WM	2 None
	Carrier (2)	44‐74	n appl	n appl	1 n/s	None	1 normal; 1 na	1 normal; 1 na	2 None
8993T>C	MILS (2)	99‐na	2 m‐3 y	1 psychomotor delay 1 subacute E + seizures	1 ID, cer/pyr 1 na (deceased at 3 y)	1 none 1 GTCS	1 slow BA 1 na	1 BG 1 BG, WM	1 None 1 na (deceased)
	NARP‐MILS (1)	97	7 y	1 A	regression, hyp, fat, mig, pyr	UOs	slow BA	BG (t), CA, WM	1 VPA
	Incomplete NARP (2)	29‐95	na	2 N	1 pyr; 1 none	2 none	2 na	2 na	2 None
8858G>A^1^	NARP‐MILS (1)	34	6 y	RP	hyp, fat, M, pyr	none	n/s	WM, CA, BG (t)	None

A, ataxia; AF, Atrial Fibrillation; As, Atonic seizures; ASM, Anti Seizure Medication; BA, background activity; BDZ, Benzodiazepines; BG, basal ganglia lesions (hyperintense signal on T2‐weighted, hypointense on T1‐weighted MRI); CA, cerebellar±cerebral atrophy; CBD, cannabidiol; cer: cerebellar signs; E, encephalopahty; ext, extrapyramidal signs; fat, fatigue; GTCS, Generalized Tonic‐Clonic Seizures; HL, heteroplasmy load %; hyp, hypoacusia; ID, intellectual disability; LEV, levetiracetam; m, months; M, Myoclonias; mig: migraine; MILS, Maternally inherited Leigh's syndrome; Ms, Myoclonic seizures; n appl, not applicablen/s: not specific EEG abnormalities; N, neuropathy; na, not available; NARP, neuropathy, ataxia, retinitis pigmentosa; PB, phenobarbital; PME, Progressive Myoclonic Epilepsy; pyr, pyramidal signs; RP, retinitis pigmentosa; sW, runs of sharply‐contoured slow waves; S‐W, spike‐wave discharges; (t), transient; THC, tetrahydrocannabinol; UOs, unknown onset seizures; VPA, valproate; WM, white mater hyperintensities; y, years.

^1^ *de novo*, never reported.

Genetic analysis highlighted the m.8993T> G mutation in 11 individuals (64.7%), 10 belonging to three families and one sporadic case (pedigrees 1‐4). Among them, nine were affected (two had MILS, four NARP‐MILS, one NARP and two incomplete NARP; HL between 48%‐98%) and two were asymptomatic carriers (HL 44%‐74%). Epileptic seizures occurred in all the four patients with NARP‐MILS, while EEG abnormalities were detected in six out of the eight individuals with available EEG, including two without seizures (Table 1).

Five affected patients from two families (pedigree 5‐6) disclosed the *MT‐ATP6* m.8993T> C mutation (HL 29%‐99%). Of them, two patients with MILS and NARP‐MILS had GTCS and brief seizures with impaired awareness, respectively. BA slowing was recorded in two patients (EEG not available in three).

Last, a novel m.8858G> A variant was found *de novo* in a sporadic case with NARP‐MILS (HL 34%, pedigree 7) without seizures neither EEG changes (Table 1).

The HL could not be assessed in the two individuals deceased (1/III.1; 6/II.1). Overall, epileptic seizures occurred in 37.5% of patients with severe phenotype (6/16, information not available in one patient deceased) carrying the m.8993T> G or the m.8993T> C mutation. The mean age at seizure onset was 8‐9 ± 9.4 years. Seizures, associated with psychomotor deterioration and/or alteration of consciousness during hyperthermia, were the presenting manifestation in two individuals. Four patients had rare GTCS alone (2) or associated with myoclonic (1) or atonic seizures (1). One patient had progressive myoclonus epilepsy (PME), one experienced brief seizures with impaired awareness.

EEG and/or polygraphic records were available in 11 patients (four with seizures, seven without). EEG abnormalities were detected in eight individuals (72.7%) with MILS (2), NARP‐MILS (4), NARP (1) or incomplete NARP (1). Half of the patients with abnormal EEG had never had seizures after a mean follow‐up of 14.5 ± 22.5 years. Two of them showed epileptic discharges, possibly associated with bursts of monomorphic slow waves (Fig 1B‐a, b), one had a burst of high‐amplitude, delta waves and one slow BA.

Surface EMG documented myoclonic bursts associated with epileptiform discharges in individual 2/II.1 (Fig 1B‐c).

QEEG analysis was performed in six affected individuals, five carrying the m.8993T> G variant and one the m.8858G> A. Their features are detailed in Appendix S2.

Statistical analysis showed a correlation between HL and disease severity (Rho = 0.63, *P* = 0.012), with a stronger correlation considering only the most frequent mutation, the m.8993T> G (Rho = 0.76, *P* = 0.011; Table 2). Seizure occurrence and the presence of EEG abnormalities were associated with a significantly higher HL (median 97% versus 81%, *P* = 0.014; median 95.5% versus 44%, *P* = 0.014). These findings are confirmed in the m.8993T> G subgroup analysis (Table 3). EEG abnormalities severity score showed a significant correlation with HL for patients carrying the m.8993T> G (Rho = 0.73, *P* = 0.040), with only a trend considering all the mutations (Rho = 0.52, *P* = 0.099). Similarly, on qEEG we found a trend towards positive correlations between HL and the AR (Rho = 0.75, *P* = 0.084). On qEEG spectral analysis, no noteworthy findings emerged, except for a weak, positive correlation between HL with SRP of delta band (Table 4; Appendix S3).

**Table 2:** Correlation between heteroplasmy load (HL, %), disease severity and EEG abnormalities score in the all variants and in the 8993T>G only

Heteroplasmy load	Spearman’s Rho	Number	*P*‐value
disease severity score – all variants	0.63	15	**0.012**
disease severity score – only 8993T>G	0.76	10	**0.011**
EEG abnormalities score – all variants	0.52	11	0.099
EEG abnormalities score – only 8993T>G	0.73	8	**0.040**

Statistically significant values (*P* values < 0.05) in bold.

**Table 3:** Comparison of HL median level and Interquartile Range (IQR) between presence (or not) of EEG abnormalities and seizures in the all variants and in the 8993T>G only

Heteroplasmy load	Median (IQR)	Number	*P*‐value
EEG abnormalities – all variants:			
Yes	95.5 (90–97.5)	8	**0.014**
No	44 (34–48)	3	
EEG abnormalities – only 8993T>G:			
Yes	93 (90–97)	6	**0.046**
No	46 (44–48)	2	
Seizures – all variants:			
Yes	97 (97–97)	5	**0.014**
No	81 (44–90)	10	
Seizures – only 8993T>G:			
Yes	97 (96.5–97.5)	4	**0.010**
No	81 (48–90)	6	

Statistically significant values (*P* values <0.05) in bold.

**Table 4:** Correlation between heteroplasmy load (%) and abnormalities ratio and sub‐band spectral relative power in six patients undergone standardized EEG protocol.

Heteroplasmy load	Spearman’s Rho	*P*‐value
Abnormalities/background ratio	0.75	0.084
Delta relative	0.49	0.32
Theta relative	0.46	0.35
Alfa relative	0.41	0.42
Beta relative	‐0.41	0.43
Gamma relative	‐0.46	0.35

## Discussion

This study focused on the epilepsy phenotype of patients with *MT‐ATP6*‐related MILS and NARP syndrome and investigated the correlation of mutant HL with seizures, EEG, and qEEG findings.

The prevalence of epileptic seizures in our case series was 37.5%, in line with literature data.^6,8^ Epilepsy occurs in patients with a severe phenotype (MILS or NARP‐MILS), most carrying the m.8993T> G change. Similarly, in a large published cohort of patients with different *MT‐ATP6* mutations, this mutation was associated with a significant higher proportion of seizures and a more severe course.^6^

Generalized seizures represent the most common seizure type; notably, one patient showed PME. Among mitochondrial disorders, PME is a recognized manifestation of MERRF (myoclonic epilepsy with ragged red fibres)^13^ whereas it is extremely rare in NARP syndrome. In fact, to date only another individual with NARP‐MILS carrying the 8993T> G change^14^ has been reported, besides our patient.

**Figure 2:**
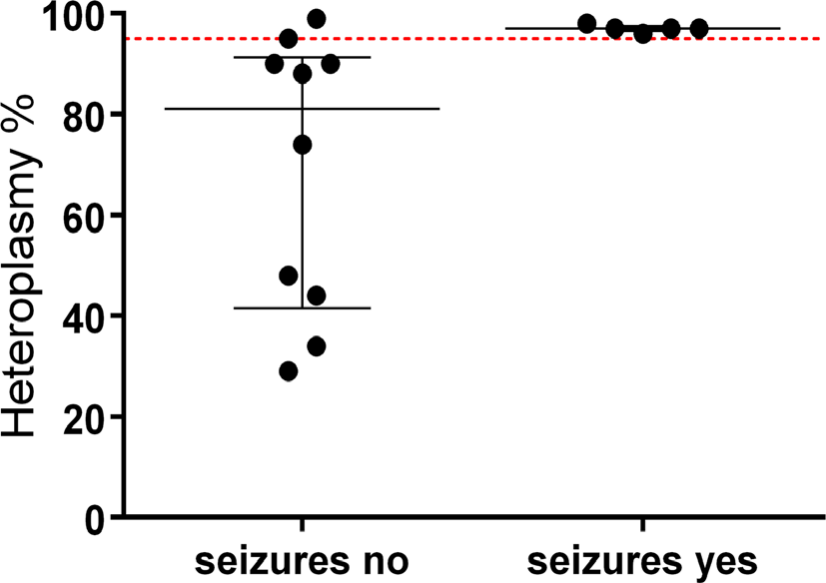
Correlation between heteroplasmy load and seizures occurrence. Kruskal–Wallis test showing a median level (and Interquartile Range, IQR) of heteroplasmy load significantly higher between the category defined by the occurrence (or not) of seizures. The dashed, red line corresponds to the best HL threshold for seizure

Statistical analysis confirmed a correlation between disease severity and HL, especially for the m.8993T> G mutation (rho = 0.76, *P* = 0.011). HL correlated also with seizure occurrence for all mutations (*P* = 0.014), thus confirming epilepsy phenotype as an index of disease severity and further highlighting that the proportion of mtDNA mutation in peripheral tissues faithfully reflects the HL distribution in the brain. The best HL threshold for seizure occurrence was set at> 95% (sensitivity 100%, specificity 90%; Fig. 2).

In our series, up to 50% of affected individuals without seizures showed EEG abnormalities (from slow BA to variable combinations of paroxysmal/epileptiform activities). Remarkably, among them even those showing epileptiform discharges have never experienced seizures after a long‐term follow‐up. This observation does not support the role of EEG abnormalities in predicting seizures, as reported.^10^ HL correlated with the presence of any EEG abnormalities (*P* = 0.014) with only a trend towards a correlation with the EEG qualitative severity score and the AR (i.e. the amount of paroxysmal activity including both, delta bursts and epileptiform discharges). Generalized background slowing and bursts of slow paroxysmal activities are common in mitochondrial diseases^15^ but no qEEG studies are available. QEEG studies on neurodegenerative diseases demonstrated a correlation of theta/delta band powers with cognitive deterioration.^16^ Thus, we interpreted the correlation of HL with EEG abnormalities (not necessarily epileptiform) and the weak correlation of HL with the relative delta power at SPR analysis, as expression of the severity of the underlying encephalopathy in patients with higher HL. Thus, as no reliable biomarkers of mitochondrial disease severity are currently available, EEG could represent a useful tool to assess disease severity, especially in patients with the m.T8993G mutation. QEEG studies on larger samples with long follow‐up, possibly including other mutational categories and clinical phenotypes, could confirm a role of relative delta power as a biomarker in mitochondrial diseases, useful to evaluate natural history and assess therapeutic efficacy in multicenter clinical trials.

## Conflicts Of Interest

None of the authors has any conflict of interest to disclose.

## Supporting information


**Appendix S1.** Supplemental Methods for SNaPshot assay, EEG standardized protocol, EEG qualitative severity score and EEG quantitative Analysis
**Appendix S2.** Supplemental Table: clinical features of the six patients with EEG quantitative study
**Appendix S3.** Supplemental Figure: Scalp EEG topographic band maps in three individuals with different heteroplasmy load (%).
