# Supplementary material for: Epilepsy in MT‐ATP6 ‐ related mils/NARP: correlation of elettroclinical features with heteroplasmy
Source: Ann Clin Transl Neurol. 2021 Jan 21;8(3):704–10. doi: 10.1002/acn3.51259 (PMC7951109; doi:10.1002/acn3.51259)
Supplement: Supplementary file 1 — Appendix S1. Supplemental Methods for SNaPshot assay, EEG standardized protocol, EEG qualitative severity score and EEG quantitative Analysis Appendix S2. Supplemental Table: clinical features of the six patients with EEG quantitative study Appendix S3. Supplemental Figure: Scalp EEG topographic band maps in three individuals with different heteroplasmy load (%). [file ACN3-8-704-s001.docx]

**APPENDIX 1**

**Supplemental Methods**

*SNaPshot assay*

Mutations were quantified using SNaPshot Multiplex System, a primer extension-based method consisting in a single base extension reaction with fluorescent labeled dideoxynucleotides, as described by Alvarez-Iglesias et al. 2008 (Doi: 10.1186/1471-2350-9-26), with minor modifications. First, the MT-ATP6 region were amplified with specific primers (forward 5’-AATGCCCTAGCCCACTTCTT-3’, reverse 5’- AGGTGGCCTGCAGTAATGTT-3’). The PCR product was purified using Agencourt AMPure XP Beads1.8X (Beckman Coulter). Single base extensions were carried out using the SNaPshot™ Multiplex Kit (ThermoFischer Scientifics), according to the manufacturer’s instructions, using specific extension primer 5’-TCAGCCTACTCATTCAACCAATAGCCC-3’ for m.8993T>G and m.8993T>C mutations, and 5’-CTAGCCATGGCCATCCCCTTATGAGCGG-3’ for m.8858G>A mutation. SNaPshot products were then purified using Agencourt AMPure XP Beads1.8X (Beckman Coulter) and 1 μL of the labeled products was mixed with 8.8 μL of Injection Buffer (Millipore) and 0.2 μL of Genescan-120LIZ size standard (Thermofisher Scientific). They were then separated on an ABI 3500Dx DNA Genetic Analyzer with POP-7 matrix. The analysis was carried out using GeneMapper ID software version 5.0 (Applied Biosystems). The percentage of mutation’s heteroplasmy was calculated as peak area ratio.

*EEG standardized protocol*

EEG was recorded during post-prandial period. Each patient underwent 40-minute recording which includes 15 minutes of resting state recording, 10 minutes of Intermittent Photic Stimulation and 5 minutes of hyperventilation (HV) and 10 minutes of post-HV period.

State of vigilance was continuously monitored by an EEG technician who by verbal alerts prevented patients from falling asleep. Nineteen scalp electrodes positioned according to the International 10-20 system referenced to C3-C4 virtual electrode were used for the recordings. Additional polygraphic electrodes were used to monitor heart and respiratory rate; bipolar surface EMG electrode were placed on left first interosseous muscle and flexor carpi radialis to record any spontaneous activities. EEG and polygraphic electrodes were sampled at 1000 Hz, impedance was kept below 5 KΩ, EEG was bandpass at 1.6-70 Hz, ECG was bandpass 0.03-35 Hz, thoracic piezoelectric transducers 1-35 Hz and surface EMG 0.003-200 Hz.

*EEG qualitative severity score*

The EEG patterns were categorized according to a severity score ranging from : 0= normal trace/mild non-specific changes; 1= slow background activity (BA); 2= slow BA and bursts of sharply-contoured slow-waves; 3=spike-wave discharges (S-W) ; 4= bursts of slow-waves and S-W; 5= slow BA, burst of slow-waves, S-W.

*EEG quantitative Analysis*

The EEG recordings were reviewed off-line, using BrainVision Analyzer (Brain Products GmbH). An expert neurophysiologist selected manually a variable number of non-overlapping, artifact-free epochs of minimum 4 seconds, depending on the amount of artifact of each recording. EEG-segments containing abnormal EEG activity (slow wave, epileptiform activity) were manually marked in artifact-free epochs.

A ratio between segments containing EEG abnormalities and artifact-free epochs was obtained for each patient (EEG abnormalities Ratio).

Fast Fourier Transform (FFT)-based spectral analysis was used to compute absolute power for the following frequency band: Delta (2-3.9 Hz), Theta (4-7.9 Hz), Alpha (8-12 Hz), Beta (13-30). Hanning windowing function without phase shift was used for the analysis at a frequency resolution of 1 Hz. Absolute power was computed by averaging the spectral power of each epoch, producing an averaged FFT power spectrum for each frequency band and electrode. Relative power, defined as the ratio between absolute power of each frequency band and the sum of overall spectral power, was computed for each electrode and expressed as a percentage. Finally, averaged relative power of overall electrodes was obtained for each frequency band.

**Appendix 2- Supplemental Table:** clinical features of the six patients with qEEG study

| **Individual** | **Sex/**  **Age(y)** | ***MT-ATP6*mutation** | **HL** | **Clinical score*** | **Age at onset** | **Regression/ psycomotor delay** | **N** | **A** | **RP** | **Seizures**  **(type)** | **EEG score*** | **ASM** |
| --- | --- | --- | --- | --- | --- | --- | --- | --- | --- | --- | --- | --- |
| 1/II.3 | M/45 | 8993T>G | 88% | 3 | 29 y | - | + | + | + | - | 2 | - |
| 2/I.2 | F/58 | 8993T>G | 48% | 2 | 32 y | - | - | + | + | - | 0 | - |
| 2/II.1 | M/35 | 8993T>G | 98% | 4 | 7 m | + | + | + | + | + PME | 5 | LEV, CBD+THC |
| 2/II.2 | F/24 | 8993T>G | 90% | 2 | 17 y | - | + | - | + | - | 3 | - |
| 4/II.1 | F/52 | 8993T>G | 90% | 5 | 2 m | + | + | + | + | - | 4 | - |
| 7/II.1 | M/46 | 8858G>A | 34% | 4 | 6 y | - | + | + | + | - | 0 | - |

**Abbreviations**: HL: heteroplasmy load (%); N: neuropathy; A: ataxia; RP: retinitis pigmentosa; ASM: anti-seizure medication; PME: Progressive Myoclonic Epilepsy;

LEV: levetiracetam; CBD: cannabidiol; THC: tetrahydrocannabinol; y: years; m: months

*Clinical severity score: 0= asymptomatic carrier;

1=incomplete NARP with 1 symptom of the classic triad;

2=incomplete NARP with 2 symptom of the triad;

3=NARP;

4= NARP associated with encephalopathy and/or transient lesions of basal ganglia (NARP-MILS);

5= MILS.

*EEG score: 0= normal/mild non-specific changes (theta slowing);

1= slow background activity (BA);

2= slow BA +bursts of sharply-contoured slow waves;

3= spike-wave (S-W) discharges;

4= burst of sharply-contoured slow waves +S-W discharges;

5= slow BA + burst of sharply-contoured slow- waves + S-W discharges

**Appendix 3 (Supplemental Figure)**


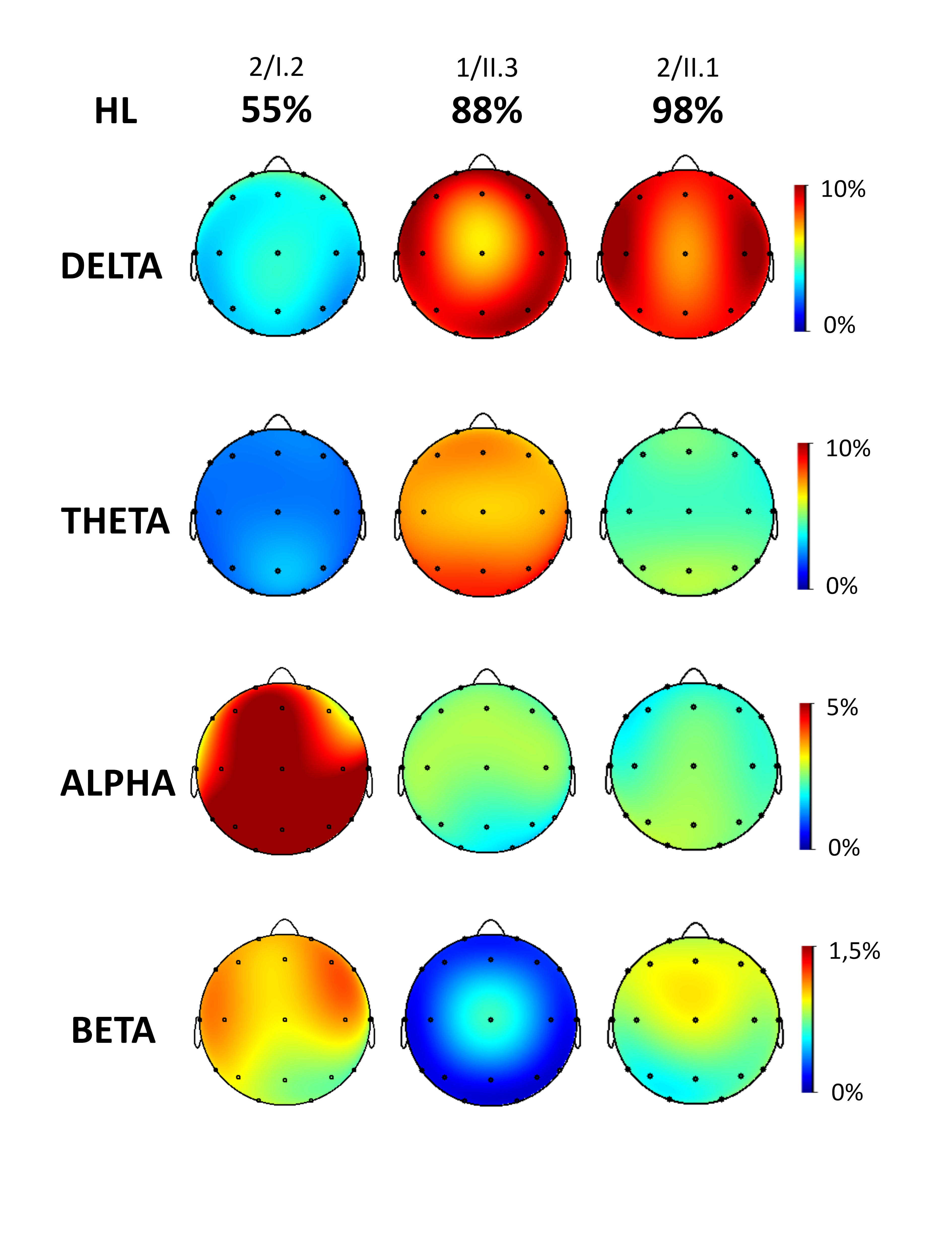
Scalp EEG topographic band maps represent the proportion of relative power for each band in three individuals with different heteroplasmy load (%).

Note that delta band relative power increases linearly with the incresing of heteroplasmy load
